# Sodium-driven energy conversion for flagellar rotation of the earliest divergent hyperthermophilic bacterium

**DOI:** 10.1038/srep12711

**Published:** 2015-08-05

**Authors:** Norihiro Takekawa, Masayoshi Nishiyama, Tsuyoshi Kaneseki, Tamotsu Kanai, Haruyuki Atomi, Seiji Kojima, Michio Homma

**Affiliations:** 1 Division of Biological Science, Graduate School of Science, Nagoya University, Chikusa-ku, Nagoya 464-8602, Japan; 2 The HAKUBI Center for Advanced Research/Institute for Integrated Cell-Material Sciences (WPI-iCeMS), Kyoto University, Sakyo-ku, Kyoto 606-8501, Japan; 3 Department of Synthetic Chemistry and Biological Chemistry, Graduate School of Engineering, Kyoto University, Nishikyo-ku, Kyoto 615-8510, Japan

## Abstract

*Aquifex aeolicus* is a hyperthermophilic, hydrogen-oxidizing and carbon-fixing bacterium that can grow at temperatures up to 95 °C. *A. aeolicus* has an almost complete set of flagellar genes that are conserved in bacteria. Here we observed that *A. aeolicus* has polar flagellum and can swim with a speed of 90 μm s^−1^ at 85 °C. We expressed the *A. aeolicus mot* genes (*motA* and *motB*), which encode the torque generating stator proteins of the flagellar motor, in a corresponding *mot* nonmotile mutant of *Escherichia coli*. Its motility was slightly recovered by expression of *A. aeolicus* MotA and chimeric MotB whose periplasmic region was replaced with that of *E. coli*. A point mutation in the *A. aeolicus* MotA cytoplasmic region remarkably enhanced the motility. Using this system in *E. coli*, we demonstrate that the *A. aeolicus* motor is driven by Na^+^. As motor proteins from hyperthermophilic bacteria represent the earliest motor proteins in evolution, this study strongly suggests that ancient bacteria used Na^+^ for energy coupling of the flagellar motor. The Na^+^-driven flagellar genes might have been laterally transferred from early-branched bacteria into late-branched bacteria and the interaction surfaces of the stator and rotor seem not to change in evolution.

In nature, many microorganisms exist that live in harsh environments. The phylum *Aquificae* includes Gram-negative and thermophilic bacteria that live in hot springs or near submarine volcanoes. Members of *Aquificae* are chemoautotrophs that oxidize hydrogen gas and use carbon dioxide as the sole carbon source^1^,^2^,^3^. Phylogenetic analysis of 16S ribosomal RNA sequences suggests that *Aquificae* diverged at an early stage in the evolutional lineage of bacteria^4^,^5^, although analyses of some individual protein sequences showed that *Aquificae* might be located near other phyla such as ε-proteobacteria^6^. *Aquifex aeolicus* is a typical *Aquificae* bacterium, and grows within a temperature range of 67–95 °C (optimal temperature = 85 °C) and a pH range of 5.4–7.5 (optimal pH = 6.8). *A. aeolicus* uses thiosulfuric acid and sulfur as well as molecular hydrogen as electron donors, and uses nitrate and nitrite as well as molecular oxygen as electron acceptors. The complete genome sequence of *A. aeolicus* has been reported^7^, and a wide range of recombinant proteins from *A. aeolicus* have been targets for structural analysis. Optical and electron microscopy showed that *Aquifex pyrophilus*, an organism closely related to *A. aeolicus*, is a rod-shaped bacterium with multiple flagella at the cell pole^1^,^8^. As bacteria move to find favorable environments by using flagellar motility, flagella are one of the most important organelles for bacteria to survive through a severe natural selection process.

The flagellum is an organelle conserved throughout a wide range of bacteria. The mechanisms involved in the assembly and function of flagellum are well studied in some bacteria such as *Escherichia coli* or *Salmonella typhimurium*. Each flagellum has a long helical structure that is about 10–15 μm long, and its assembly involves more than 50 different genes^9^. The flagellum works as a screw by rotation, and its energy source is the electrochemical potential difference between the inside and outside of the cell. The flagellar motor, which is located at the base of the flagellum, is composed of a rotor and stator. The stator is a membrane-embedded energy converter made of four MotA molecules and two MotB molecules^10^,^11^ (Fig. 1A,B), and converts the ion influx through itself into the rotation of the rotor. MotA and MotB are a four TM protein and a single TM protein, respectively^12^,^13^ (Figs 1B and S2). The third and fourth TM segments of MotA and the TM segment of MotB is thought to form an ion conductive pathway^11^,^14^,^15^. The stator is separated into three parts in terms of function, i) a large periplasmic region which contains a Peptidoglycan (PG)-binding domain and changes its conformation dynamically to anchor to the PG layer to fix the stators around the rotor^16^,^17^, ii) the plug and transmembrane (TM) region which regulates the ion conductivity and forms a selective ion channel^18^,^19^, and iii) a large cytoplasmic region which is important for the assembly around the rotor and directly interacts with the rotor (FliG) to generate the rotational force^20^,^21^,^22^.

The coupling ions of the stator can differ among various bacteria. For example, *E. coli* and *S. typhimurium* have H^+^-driven stators^23^, and *Vibrio alginolyticus* and *Vibrio parahaemolyticus* have Na^+^-driven stators for their polar flagellum and H^+^-driven stators for lateral flagella^24^,^25^. *Bacillus subtilis* and *Shewanella oneidensis* also have both H^+^- and Na^+^-driven stators, but both function to drive a single rotor^26^,^27^,^28^. The alkalophilic bacterium *Bacillus alcalophilus* has a stator which conducts both Na^+^ and K^+^ 29. When the periplasmic region of the Na^+^-driven stator from *V. alginolyticus* was replaced with the periplasmic region of *E. coli*, the hybrid stator could rotate the rotor of the *E. coli* flagellar motor^30^. The periplasmic region of the stator is properly optimized in each bacterial species or particular flagellar system whereas the TM and cytoplasmic regions of the stator are exchangeable between *E. coli* and *V. alginolyticus*. A similar exchangeability of the domains of the stator was also reported between those in *V. alginolyticus* and *Rhodobacter sphaeroides*^31^.

In this study, we focused on the formation and the function of flagella of the hyperthermophilic bacterium, *A. aeolicus.* We cultured this hyperthermophilic bacterium and observed its flagellum and motile behavior. From this bacterium, we cloned the genes for the stator and found that the stator of *A. aeolicus* could be compatible with the native stator of *E. coli*, by replacement of the periplasmic region of the *A. aeolicus* stator with that of *E. coli*. Based on our results, we discuss the thermo-stabilized feature of the flagellar system of hyperthermophilic bacteria, the ion selectivity and universal function of the stator units as torque generators in the motor, and the phylogenetic relationship of stators among bacterial species.

## Results

### Characterization of *A. aeolicus* flagella and motility

*A. aeolicus* has almost all of the flagellar genes conserved in Gram-negative bacteria (Fig. S1). However, there has been no report showing whether *A. aeolicus* actually has flagella. To examine this point, we cultivated the wild-type *A. aeolicus* VF5 strain in inorganic medium under a H_2_/CO_2_/O_2_ gas atmosphere at its optimum growth temperature of 85 °C, and the cultivated cells were observed by transmission electron microscope (TEM) using negative staining. The cells were rod-shaped and flagellar filaments existed at the pole of some cells (Fig. 2A,B), although most of the cells did not have flagella. The most of flagellated cells have a single polar flagellum, while few cells harbored multiple flagella at their cell pole (Fig. 2C).

Next, we examined whether *A. aeolicus* could swim in solution. We enclosed culture broth to a variable–temperature chamber for optical microscopy (Fig. 3A), and then monitored the cells at different temperatures. At a temperature of 85 °C, we observed that only a low population of cells swam smoothly (Movie S1), while most of the cells did not show motility, consistent with the TEM observation result that most cells did not have flagellar filaments. The swimming speed of the cells was 93 ± 40 μm s^−1^ (mean ± SD, *n* = 55) at 85 °C (Fig. 3B,C). Most of the cells swam linearly, however, a few cells showed changes in direction during their swimming. The swimming speed decreased with decreasing temperature of the chamber, and exhibited 16 ± 5 μm s^−1^ (mean ± SD, *n* = 90) at room temperature (22 °C) (Fig. 3C). These results indicate that the motility machinery, presumably the flagellum, of *A. aeolicus* functions well at high-temperature conditions and needs high-temperature for its maximum function.

### The flagellar stator proteins of *A. aeolicus*

*A. aeolicus* harbors one *motA* and two *motB* genes that constitute an operon (*motA*^*Aa*^/*motB*_*1*_^*Aa*^/*motB*_*2*_^*Aa*^)^7^, whereas other bacteria commonly have one set (one *motA*/*motB* gene) or two sets (two *motA*/*motB* genes) of stator genes. The MotA protein of *A. aeolicus* (MotA^Aa^) shares 30.5% sequence similarity with MotA of *E. coli* (MotA^Ec^). MotB_1_^Aa^ and MotB_2_^Aa^ share 35.6% and 36.3% sequence similarity with MotB^Ec^, respectively. Conserved charged residues mainly important for stator-rotor interaction, R90 and E98 in MotA^Ec^, are conserved as R88 and E96 in MotA^Aa^. The secondary important charged residue, E150 in MotA^Ec^, is not conserved in MotA^Aa^ (Fig. S2A). When the sequences of the two MotB proteins of *A. aeolicus* are compared, the TM and N-terminal regions are very similar (sequence identity is 97%) except for one residue (Gly^20^ in MotB_1_^Aa^ is Ser^20^ in MotB_2_^Aa^), whereas the C-terminal periplasmic region is vastly different (sequence identity is 23.0%) (Fig. S2B). When the predicted secondary structures of MotB^Ec^, MotB_1_^Aa^ and MotB_2_^Aa^ are compared, the flexible linker sequences between the plug region and the OmpA-like domain, which works as a PG-binding domain, are very different (Fig. 4A), and their linker lengths are 82, 70 and 56, respectively.

### Chimeric stators of *A. aeolicus* function in *E. coli*

To examine whether MotA/MotB_1_ or MotA/MotB_2_ of *A. aeolicus* have the ability to rotate the flagellar rotor of *E. coli*, we cloned their genes from the chromosomal DNA and introduced those genes into nonmotile *E. coli* Δ*motAB* cells and checked their motility in soft-agar plates at 30 °C. We found that introduction of MotA^Aa^/MotB_1_^Aa^ or MotA^Aa^/MotB_2_^Aa^ did not restore the motility of *E. coli* Δ*motAB* cells (Fig. 4B). We constructed two chimeric genes to produce chimeric MotB proteins whose N-terminal TM regions of MotB_1_^Aa^ or MotB_2_^Aa^ were fused to the C-terminal periplasmic region of MotB^Ec^ (Fig. 4A). The two hybrid stators, named MotA^Aa^/MotB_1_^AE^ and MotA^Aa^/MotB_2_^AE^, conferred slight outspreading of the *E. coli* cells in soft-agar plates (Fig. 4B,C). This indicates that the hybrid stators can function in *E. coli*.

After a long incubation of *E. coli* cells expressing MotA^Aa^/MotB_2_^AE^ in soft-agar plates, we obtained a spontaneous up-motile mutant that showed a larger swimming ring (Fig. 4C). From this mutant, we found a point mutation near the 3’ -end of the *motA*^*Aa*^ gene, which leads to an amino acid substitution of A225D (GCT - > GAT). Under the microscope, the swimming speeds of these *E. coli* producing chimeric stators were very slow (around 10 μm s^−1^ at maximum for the A225D mutant while wild-type *E. coli* swims at around 30 μm s^−1^) and most cells only jiggled in place without fast locomotion (Fig. S3, Movie S2). Changing the observation temperature (20–45 °C) did not strongly affect the swimming of chimeric *E. coli* cells (Movie S3).

To investigate the function of the chimeric stators for the rotation of a single motor, we performed the tethered cell assay. We attached a flagellar filament to a cover glass and the fractions and speeds of rotation of the *E. coli* cells were measured (Movie S4). Regarding the MotA^Aa^-A225D mutation, the *E. coli* cells expressing chimeric MotA^Aa^/MotB^AE^ increased the fractions of rotation but not the speed (Fig. S4A and B). The rotation speeds of *E. coli* cells expressing chimeric stators decreased with lower concentrations of NaCl in the buffer and the rotations completely stopped in the absence of NaCl (Fig. 5A,B). This indicates that the stator of *A. aeolicus* is a Na^+^-driven stator. The Michaelis constants (Km) for Na^+^ are 12 ± 2 mM or 13 ± 4 mM in the *E. coli* cells expressing chimeric MotA^Aa^/B_1_^AE^ or MotA^Aa^/B_2_^AE^, respectively. Those values are a little higher than the previously reported Km of other Na^+^-driven flagellar motor (ca. 2.2 mM)^32^. In contrast, the rotation speed of *E. coli* cells expressing native MotA^Ec^/MotB^Ec^ was not affected by the NaCl concentration (Fig. 5C).

## Discussion

There are many kinds of organisms that adapt their biological activities and life styles to the environment where they live, and their morphologies, organelles and biomolecules are modulated to function in their specific environmental conditions. Especially, extremophiles, organisms living in physically and/or chemically extreme conditions, seem to have extremely specialized molecules to survive in harsh environments. Here, we focused on the flagella, which have one of the most important roles for survival strategy of bacteria, of the hyperthermophilic bacterium, *A. aeolicus*.

We showed that flagellated *A. aeolicus* cells mainly have a single flagellum at the cell pole, although *A. pyrophilus*, a close relative of *A. aeolicus*, was reported to have multiple flagella at the cell pole^1^,^8^. It was reported that the marine bacterium *V. alginolyticus* has a single polar flagellum, and that two cytoplasmic proteins, FlhF and FlhG, regulate the numbers of the flagella^33^,^34^. *A. aeolicus* also has the *flhF* and *flhG* genes. We speculate that FlhF and FlhG regulate the flagellar number and location for a single polar flagellum of *A. aeolicus*. We revealed that *A. aeolicus* displays a temperature-dependent motility in solution: its swimming speed was highest at 85 °C, and decreased in response to reductions in temperature, which is similar to a previous report on the motility of another thermophilic bacterium, *Thermotoga maritima*^35^. Most *A. aeolicus* cells observed in this study swam linearly. *A. aeolicus* may not change the direction of flagellar rotation because it does not have homologues of the *che* receptor genes and the *fliM* gene of the switch protein.

The function of the flagellum has been well studied in *E. coli* and various experimental systems have been established. We introduced the stator proteins MotA and MotB, which are the two most important proteins for the energy transduction, of *A. aeolicus* into *E. coli*. Previous analyses of the MotA/MotB stator units indicate that this unit works as an ion channel, and it is thought that the ion influx causes the dynamic conformational change of cytoplasmic region of the stator to generate a torque^36^. We showed that the whole stator unit of *A. aeolicus* could not replace the native MotA/MotB stator of *E. coli*, but the chimeric stator, in which the periplasmic region of MotB_1_^Aa^ or MotB_2_^Aa^ is replaced with the corresponding region of MotB^Ec^, could function in *E. coli*. The periplasmic region of MotB is believed to change its conformation and to bind the PG layer for the proper function of the stator^16^,^17^. The periplasmic regions of *A. aeolicus* stators probably cannot anchor the stator around the rotor of *E. coli* because of the poor compatibility with the *E. coli* PG layer. This region of *E. coli* is important for the correct assembly of the stator around the rotor in *E. coli*^16^,^37^.

**Figure 1:**
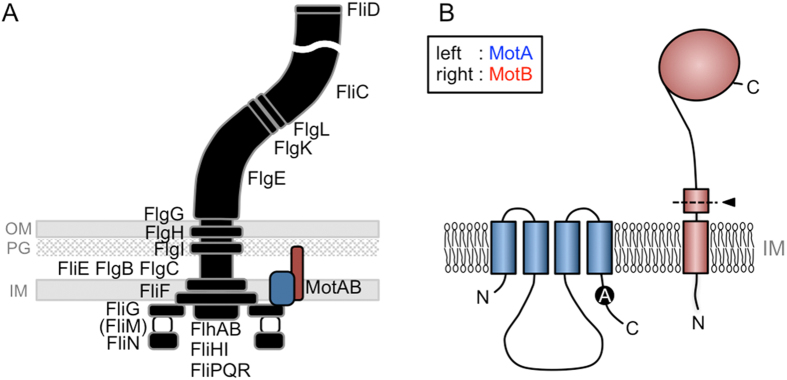
Schematic cartoon of the flagellum and the stator proteins. (**A**) The flagellum is a large complex composed of many proteins and consists of a filament, a hook and a basal body. *A. aeolicus* has most genes for flagellar component except for FliM. (**B**) The stator is composed of two membrane proteins, MotA (blue) and MotB (red). MotA is a four TM protein and MotB is single TM protein. The spontaneous mutated Alanine residue is indicated as ‘A’ in the black circle. Black arrowhead, border of chimeric MotB: OM, outer membrane; PG, peptidoglycan layer; IM, inner membrane.

**Figure 2:**
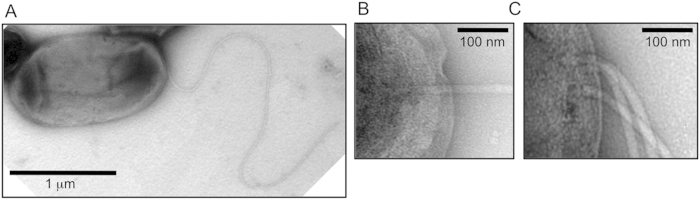
Transmission electron micrograph of the cells of *A. aeolicus*. (**A**) Image of the whole cell and its flagellum. (**B**) and (**C**) Partial enlargements of the flagellated cell pole. Cells were negatively stained with 1% uranyl acetate.

**Figure 3:**
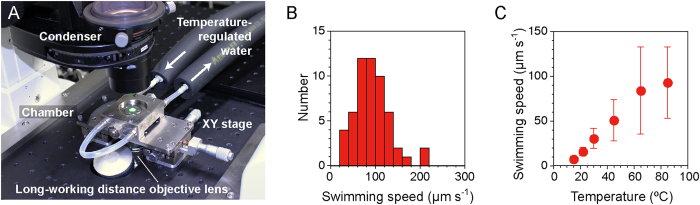
Swimming speed of *A. aeolicus* in solution. (**A**) Photograph of the variable-temperature chamber used in this study. (**B**) Histogram of swimming speed of the cells at 85 °C, the optimal growth temperature. (**C**) Thermo-dependent swimming speed of the *A. aeolicus* cells. The cells were grown at 85 °C and observed at each temperature.

**Figure 4:**
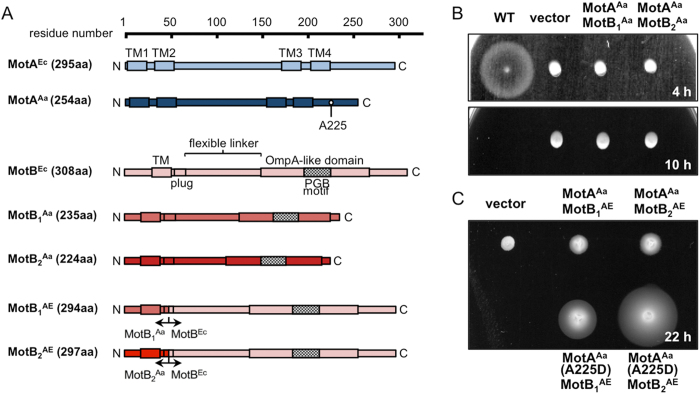
Motility assay of *E. coli* cells producing MotA and MotB of *A. aeolicus* in soft-agar plates. (**A**) Schematics of primary structures of MotA, MotB of *E. coli* and *A. aeolicus* and chimera MotB. We switched the sequence at the middle of the plug region for chimera MotB. (**B**) and (**C**) Motilities of *E. coli* cells in soft-agar plate. MotA and MotB were expressed from two compatible plasmids which were constructed from pBAD24 and pSBETa, respectively, in a *E. coli* Δ*motAB* strain. Plates were incubated at 30 °C for the indicated number of hours. ^Ec^, protein of *E. coli*; ^Aa^, protein of *A. aeolicus*; ^AE^, chimera fused proteins of *A. aeolicus* and *E. coli*.

**Figure 5:**
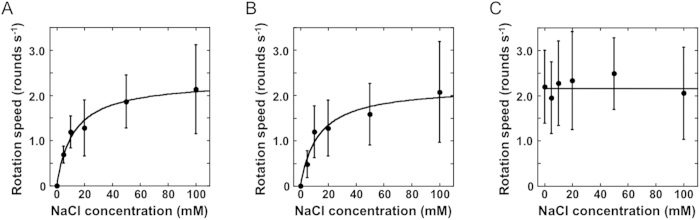
Na^+^ dependent motor function of *E. coli* cells producing MotA and chimera MotB proteins. Rotation speeds of *E. coli* (Δ*motAB*) cells producing chimeric stators with the MotA-A225D mutation (MotA^Aa^(A225D)/MotB_1_^AE^ for (**A**), and MotA^Aa^(A225D)/MotB_2_^AE^ for (**B**)) or the native *E. coli* stator (**C**) were measured in various Na^+^ concentrations as noted.

We have isolated a mutant with a single amino acid substitution in MotA^Aa^ (A225D) exhibiting an up-motile phenotype in *E. coli* expressing chimeric stators. Ala^225^ is located at the cytoplasmic C-terminal region of MotA^Aa^ (Fig. 1B), which is not a highly conserved residue among MotAs. The mutation did not increase the rotation speed but increased the fraction of the motors that rotate (Fig. S4). This may suggest that the A225D mutation in MotA^Aa^ improves the efficiency of the assembly of the stators around the rotor, leading to an increase in the ratio of the functional motors. Although it is difficult to discuss the detailed effect of the mutation because of the absence of structural information on the cytoplasmic region of MotA, we can speculate that the point mutation in the MotA^Aa^ protein provides better flexibility at low temperatures that improves the function in the *E. coli* motor at these temperatures.

**Figure 6:**
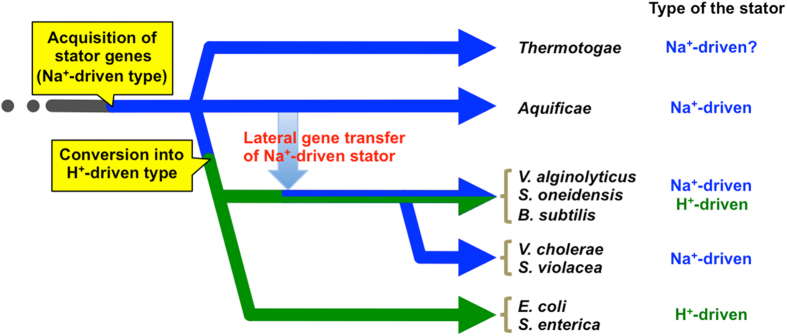
Schematic showing the acquisition and derivation of stator genes through bacterial evolution. First, the ancestral bacteria acquired the Na^+^-driven stator, and earliest branched bacteria have the Na^+^-driven stator. The stator then was converted into the H^+^-driven type through the middle period of bacterial evolution. The Na^+^-driven stator of the late-branched bacteria seems to be provided from early-branched bacteria (*Aquificae* or *Thermotogae*) by lateral gene transfer.

The fact that MotA in the flagellar motor of *E. coli* is replaceable with MotA of *A. aeolicus* indicates that the interaction surface of the stator and rotor is highly conserved not only among phylogenetically close species such as *E. coli*, *V. alginolyticus* and *R. sphaeroides* as previously reported^30^,^31^, but also between phylogenetically distant species. This may correlate with the high similarity among the crystal structures of FliG, a rotor component that directly interacts with MotA via electrostatic interaction, from *T. maritima*, *Helicobacter pylori* and *A. aeolicus*^38^,^39^,^40^. We also showed that chimeric *E. coli* FliG, whose C-terminal domain where directly interact with MotA is replaced with *A. aeolicus* FliG, is compatible with native FliG in *E. coli* (Fig. S5), supporting the conservation of the mechanism for the motor function among bacteria. The phylogenetic analysis of *motA* and *motB* genes from various bacteria suggests high sequence similarities among *motA*/*motB* (or their orthologous) genes of *A. aeolicus*, *T. maritima*, *Vibrio* species and *B. subtilis*, which mostly encode Na^+^-driven stators (Fig. S6). Currently, there are two alternative hypothetical scenarios for the evolution of *Aquificae*; i) *Aquificae* branched at an early stage in the evolutionary lineage of bacteria^4^,^5^, and ii) *Aquificae* belong to a group near the ε-proteobacteria and some genes, including ribosomal genes, are derived from other species by lateral gene transfer^6^,^41^. Whichever scenario is correct, the flagellar genes of *A. aeolicus* are thought to be among the most primordial flagellar genes, because of the sequence similarities with those of *T. maritima*, which is also thought to be an early-branched bacterium. These lines of evidence suggest that the earliest stator of the flagellar motor is a Na^+^-driven type stator. Through the evolution of bacteria, the function of the stator might have been converted into the H^+^-driven type, and the Na^+^-driven type stator might have been re-acquired in some late-branching bacteria by lateral gene transfer from *Aquificae* in some specific species (Fig. 6). The low sequence similarity between the two types of stators in a single species (e.g. MotA/B (H^+^ type) and PomA/B (Na^+^ type) in *Vibrio* or *Shewanella*, or MotA/B (H^+^ type) and MotP/S (Na^+^ type) in *Bacillus*) also supports the occurrence of lateral gene transfer of the Na^+^-driven stator.

In general, bacteria and mitochondria of eukaryotes use H^+^ for energy conversion. Our study reveals that *A. aeolicus* uses Na^+^ for energy conversion for the flagellar motor. We speculate that ancient bacteria, which mainly lived in the sea, use Na^+^ but not H^+^ for their biological activities across the cell membrane, such as the synthesis of ATP by ATP synthase or the torque generation of the flagellar motor.

## Methods

### Cultivation of *A. aeolicus* cells

*Aquifex aeolicus* was kindly provided by Dr. Harald Huber (University of Regensburg, Germany). *A. aeolicus* cells were cultivated in modified SME medium^42^, containing per liter: NaCl 30.0 g; MgSO_4_·7H_2_O 7.0 g; MgCl_2_·6H_2_O 5.5 g; KCl 0.65 g; NaBr 0.1 g; NaHCO_3_ 2.0 g; NH_4_Cl 0.15 g; K_2_HPO_4_ 0.15 g; CaCl_2_·2H_2_O 0.15 g; NaS_2_O_4_ 2.0 g; 10 ml trace mineral solution and 10 ml trace vitamin solution. The trace mineral solution was based on trace minerals of the report^43^ that contains per liter: nitrilotriacetic acid 1.5 g; MgSO_4_·7H_2_O 3.0 g; MnSO_4_·5H_2_O 0.5 g; NaCl 1 g; FeSO_4_·7H_2_O 0.1 g; CoSO_4_·2H_2_O 0.1 g; CaCl_2_·2H_2_O 0.1 g; ZnSO_4_·7H_2_O 0.1 g; CuSO_4_·5H_2_O 0.01 g; AlK(SO_4_)_2_ 0.01 g; H_3_BO_3_ 0.01 g; Na_2_MoO_4_ 0.01 g; (NH_4_)_2_Ni(SO_4_)_2_·6H_2_O 2.0 g; Na_2_WO_4_·2H_2_O 0.01 g; and Na_2_SeO_4_ 0.01 g. In order to make the trace mineral solution, nitrilotriacetic acid was first dissolved with KOH to pH 6.5, then minerals were added, and finally the pH was adjusted to 7.0 with KOH (or H_2_SO_4_). The trace vitamin solution was made to contain per liter: biotin 2 mg; folic acid 2mg; pyridoxin·HCl 5 mg; thiamine·HCl 5 mg; riboflavin 5 mg; nicotinic acid 5mg; calcium pantothenate 5 mg; vitamin B_12_ 5mg; *p*-aminobenzoic acid 5 mg; and lipoic acid 5 mg. The trace mineral and the trace vitamin solutions were sterilized by filtration (0.2 μm pore size). For media preparation, the pH of the medium was first adjusted to 6.5–6.8 with H_2_SO_4_/KOH before the addition of the trace mineral and trace vitamin solutions. This medium was then sterilized by filtration (0.2 μm pore size). Sterilized culture test tubes with butyl rubber cups were filled with 10 ml of the above medium, and each 0.1 ml of the trace mineral and the trace vitamin solution was added. After inoculation of the stock culture, the culture tubes were degassed three times and exchanged with H_2_/CO_2_ gas (80:20) to atmospheric pressure (−0.1 MPa). In order to make a micro-aerobic gas condition, air was introduced to adjust the final O_2_ concentration around 1%. The tubes were then pressurized with H_2_/CO_2_ gas (80:20) to 0.2 MPa, and they were incubated at 85 °C. In a typical case, growth was detected after incubation of 18–24 h.

### Observation of *A. aeolicus* cell by transmission electron microscopy

Wild-type *A. aeolicus* cells were grown for 10 h at 85 °C, without shaking (OD660 ~ 0.004). 10 ml of the cells were harvested by centrifugation and suspended in 3 μl of 1% uranyl acetate. The samples were put on a carbon-coated copper grid and observed with an electron microscope (JEM1011, JEOL, Japan).

### Motility assay of *A. aeolicus*

Wild-type *A. aeolicus* cells were grown for 10 h at 85 °C, without shaking (OD660 ~ 0.004). The culture medium containing grown cells was introduced into a variable-temperature chamber as described before^44^,^45^. The inner temperature of the chamber was controlled by running temperature-regulated water from a thermostat bath. Microscopic observation in the chamber was done by a long-working distance objective lens (CFI ELWD ADM 20×C), and phase-contrast images were acquired by a charge-coupled device camera (WAT-120N+, Watec, Tsurugaoka, Japan). The images of cell movement were recorded and the swimming speed of each cell was measured.

### Strains, growth conditions and media of *E. coli*

Bacterial strains used in this study are listed in Table S1. *E. coli* cells were cultured at 37 °C in LB medium [1% (w/v) bacto tryptone, 0.5% (w/v) yeast extract, 0.5% (w/v) NaCl] or at 30 °C in TG medium [1% (w/v) bacto tryptone, 0.5%(w/v) NaCl, 1% (w/v) glycerol]. If needed, kanamycin or ampicillin was added to a final concentration of 25 μg ml^−1^ or 50 μg ml^−1^, respectively.

### Cloning and construction of plasmids

Plasmids used in this study are listed in Table S1. The *motA*^*Aa*^, *motB*_*1*_^*Aa*^, *motB*_*2*_^*Aa*^, and *fliG*^*Aa*^ genes were cloned from the chromosome of *A. aeolicus*, a kind gift from Dr. Harald Huber. *motA*^*Aa*^, *motB*_*1*_^*Aa*^, *motB*_*2*_^*Aa*^ and *fliG*^*Aa*^ genes on chromosomal DNA were PCR-amplified using upstream sense primers containing a *Nde*I site and a downstream antisense primer containing a *Xba*I site for *motA*^*Aa*^ gene or a *Bam*HI site for *motB*^*Aa*^ and *fliG*^*Aa*^ genes. PCR-amplified DNA fragments and plasmid vectors, pColdI (Takara) and pSBETa, were digested with the respective restriction enzymes, and then were separated using agarose gel electrophoresis. The fragments were purified using a gel extraction kit (Qiagen) and were ligated using T4 DNA ligase to generate pNT8, pNT9, pNT12 and pNT14. The *motA*^*Aa*^ gene on pNT7 was subcloned from pNT12. The *motA*^*Aa*^ gene with SD sequence on pNT12 was PCR-amplified using an upstream sense primer containing a *Kpn*I site and a downstream antisense primer containing a *Xba*I site. The PCR-amplified DNA fragment and pBAD24, a plasmid vector, were digested with *Kpn*I and *Xba*I, purified, and ligated as described above to generate pNT7. The *fliG*^*Ec*^ gene on pNT13 was subcloned from pTY301 using a similar method through *Nde*I and *Bam*HI. The construction of chimeric genes on pNT10 and pNT11 was carried out using two-step PCR. In the first step, fragments of *motB*_*1*_^*Aa*^ and *motB*_*2*_^*Aa*^ genes on pNT8 and pNT9, respectively, were PCR-amplified using upstream sense primers containing a *Nde*I site and downstream antisense primer whose 5’ sequence is for *motB*^*Ec*^ and 3’ sequence is for *motB*^*Aa*^. Fragments of *motB*^*Ec*^ genes on pYA6022 were also PCR-amplified using upstream sense primers whose 5’ sequence is for *motB*^*Aa*^ and 3’ sequence is for *motB*^*Ec*^ and a downstream antisense primer containing a *Bam*HI site. The amplified DNA fragments were purified as described above. In the second step, the DNA fragments were mixed together, and the full-length genes, *motB*_*1*_^*AE*^ and *motB*_*2*_^*AE*^, were PCR-amplified using an upstream sense primer containing a *Nde*I site and a downstream antisense primer containing a *Bam*HI site. PCR-amplified DNA fragments and pSBETa were digested with *Nde*I and *Bam*HI, purified, and ligated as described above to generate pNT10 and pNT11. The construction of the chimeric *fliG*^*EA*^ gene on pNT15 was carried out using a similar method through *Nde*I and *Bam*HI.

### Motility assay in soft-agar plates

Two μl of an overnight culture of RP437 or RP6894 cells harboring plasmids were spotted on TB soft-agar plates [1% (w/v) Bacto Tryptone, 0.5% (w/v) NaCl, 0.25% (w/v) Bacto agar] with 0.02% (w/v) arabinose and 0.5 mM isopropyl β-_D_-1-thiogalactopyranoside (IPTG) and were incubated at 30 °C for the appropriate hours as noted in the text.

### Isolation of the spontaneous up-motile mutant

The colonies of RP6894 cells harboring the plasmid pNT10 or pNT11 were scratched on a TB soft-agar plate with 0.02% (w/v) arabinose and 0.5 mM IPTG and were incubated at 30 °C for 2 days. The motility halo was picked from the plate, plasmids were purified from the cells, and RP6849 was retransformed by the purified plasmids to check whether mutations were located in the plasmids. Sequences of the plasmids were checked by DNA sequencing.

### Tethered cell assay

Overnight cultures of YS34 cells harboring the plasmids grown in LB medium were inoculated at a 100-fold dilution into TG medium with 0.02% (w/v) arabinose and 0.5 mM IPTG and were cultured at 30 °C for 4 hours. Cells were harvested by centrifugation and were washed three times by motility buffer [10mM potassium phosphate (pH 7.0), 0.1 mM EDTA] with or without NaCl (0, 5, 10, 20, 50, 85, 100 mM). Forty μl of each cell suspension were loaded into the tunnel slides composed of a slide glass and a cover glass with a spacer, and were then inverted and incubated for 10 minutes. Another 80 μl of motility buffer were loaded into each tunnel slide to remove the remaining unattached cells. The rotation of the cells was observed using dark-field microscopy and images were acquired with a CCD camera (WV-1550, National, Japan). The images of cells were recorded and rotation speeds were measured. At the same time, rotated cells in the observed fields were counted, and those percentages to all cells in the fields are shown as the rotating fraction.

## Additional Information

**How to cite this article**: Takekawa, N. *et al.* Sodium-driven energy conversion for flagellar rotation of the earliest divergent hyperthermophilic bacterium. *Sci. Rep.* **5**, 12711; doi: 10.1038/srep12711 (2015).

## Supplementary Material

Supplementary Information

Supplementary Movie S1

Supplementary Movie S2

Supplementary Movie S3

Supplementary Movie S4
