# Supplementary material for: Sodium-driven energy conversion for flagellar rotation of the earliest divergent hyperthermophilic bacterium
Source: Sci Rep. 2015 Aug 5;5:12711. doi: 10.1038/srep12711 (PMC4525482; doi:10.1038/srep12711)
Supplement: Supplementary Information [file srep12711-s1.pdf]

Sodium-driven energy conversion for flagellar rotation of the earliest divergent hyperthermophilic bacterium

Norihiro Takekawa [1], Masayoshi Nishiyama [2], Tsuyoshi Kaneseki [3], Tamotsu Kanai [3], Haruyuki Atomi [3], Seiji Kojima [1] and Michio Homma [1]*

[1] Division of Biological Science, Graduate School of Science, Nagoya University, Chikusa-ku, Nagoya 464-8602, Japan

[2] The HAKUBI Center for Advanced Research / Institute for Integrated Cell-Material Sciences (WPI-iCeMS), Kyoto University, Sakyo-ku, Kyoto 606-8501, Japan

[3] Department of Synthetic Chemistry and Biological Chemistry, Graduate School of Engineering, Kyoto University, Nishikyo-ku, Kyoto 615-8510, Japan

*Corresponding author:
Phone: 81 52 789 2991
Fax: 81 52 789 3054
E-mail address: g44416a@cc.nagoya-u.ac.jp

Table S1. Strains and plasmids used in this study.

Strain or plasmid	Description	Source or reference
Aquifex aeolicus VF5	Wild-type *Aquifex aeolicus* strain	H. Huber
E. coli strains		
DH5α	Recipient for cloning expriments	(1)
RP437	Wild-type for motility	(2)
RP6894	RP437Δ*motAB*	J. S. Parkinson
YS34	Δ*cheY*, *fliC::Tn10*, Δ*pilA*, Δ*motAmotB*	(3)
Plasmids		
pBAD24	Amp^r P_{BAD}	(4)
pNT7	pBAD24- $motA^{Aa}$	This study
pSU41	Km^r P_{lac}	(5)
pYA6022	pSU41- $motAB^{Ec}$	(6)
pBAD33	Cm^r P_{BAD}	(4)
pTY301	pBAD33-$fliG^{Ec}$	(7)
pSBETa	Km^r P_{T7} argU	(8)
pNT8	pSBETa- $motB_1^{Aa}$	This study
pNT9	pSBETa- $motB_2^{Aa}$	This study
pNT10	pSBETa- $motB_1^{AE}$	This study
pNT11	pSBETa- $motB_2^{AE}$	This study
pNT13	pSBETa-$fliG^{Ec}$	This study
pNT14	pSBETa-$fliG^{Aa}$	This study
pNT15	pSBETa-$fliG^{EA}$	This study
pColdI	Amp^r P_{cspA} (Cold shock expression vector)	Takara
pNT12	pColdI- $motA^{Aa}$	This study

^{Aa}, genes of *A. aeolicus*; ^{Ec}, genes of *E. coli*; ^{AE} and ^{EA}, chimera genes fusing ^{Aa} and ^{Ec}; Amp^r, ampicillin resistant; Km^r, kanamycin resistant; Cm^r, chloramphenicol resistant; Strep/$Spec^r$, streptomycin/spectinomycin resistant; $P_{BAD,}$ arabinose promoter; P_{T7}, *T7* promoter; P_{cspA}, promoter of CspA, a major cold shock protein of *E. coli*. (1) Proc Natl Acad Sci USA, 87: 4645-4649. (2) J Bacteriol, 151:106-113. (3) Nature, 437: 916-919. (4) J Bacteriol, 177: 4121-4130. (5) Gene, 102:75-78. (6) J Mol Biol, 327:453-463. (7) *J Mol Biol*, 334:567-583. (8) BioTechniques, 19:196-198.

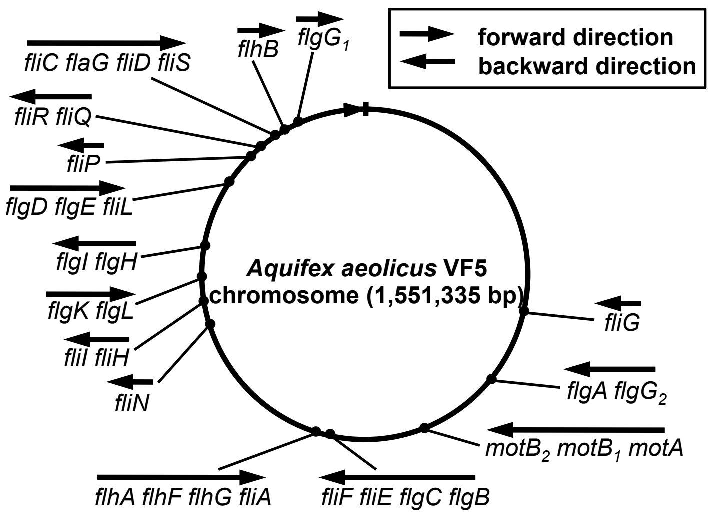

Fig. S1. Chromosomal map of flagellar genes of *A. aeolicus*. The map was drawn based on the previously reported whole genome sequence of *A. aeolicus* VF5 strain (7). The genome of *A. aeolicus* consists of a single 1.55 Mbp chromosome and the flagellar genes are dispersed throughout the chromosome as small clusters. *A. aeolicus* has almost all flagellar genes conserved in Gram-negative bacteria except for some genes coding for FliM important for directional switching of the flagellar motor, the transmembrane sensor proteins such like MCP (methyl-accepting chemotaxis protein) or MLP (mcp-like-protein), and Che proteins involved in chemotactic signaling pathway.

Fig. S1. Takekawa *et al.*

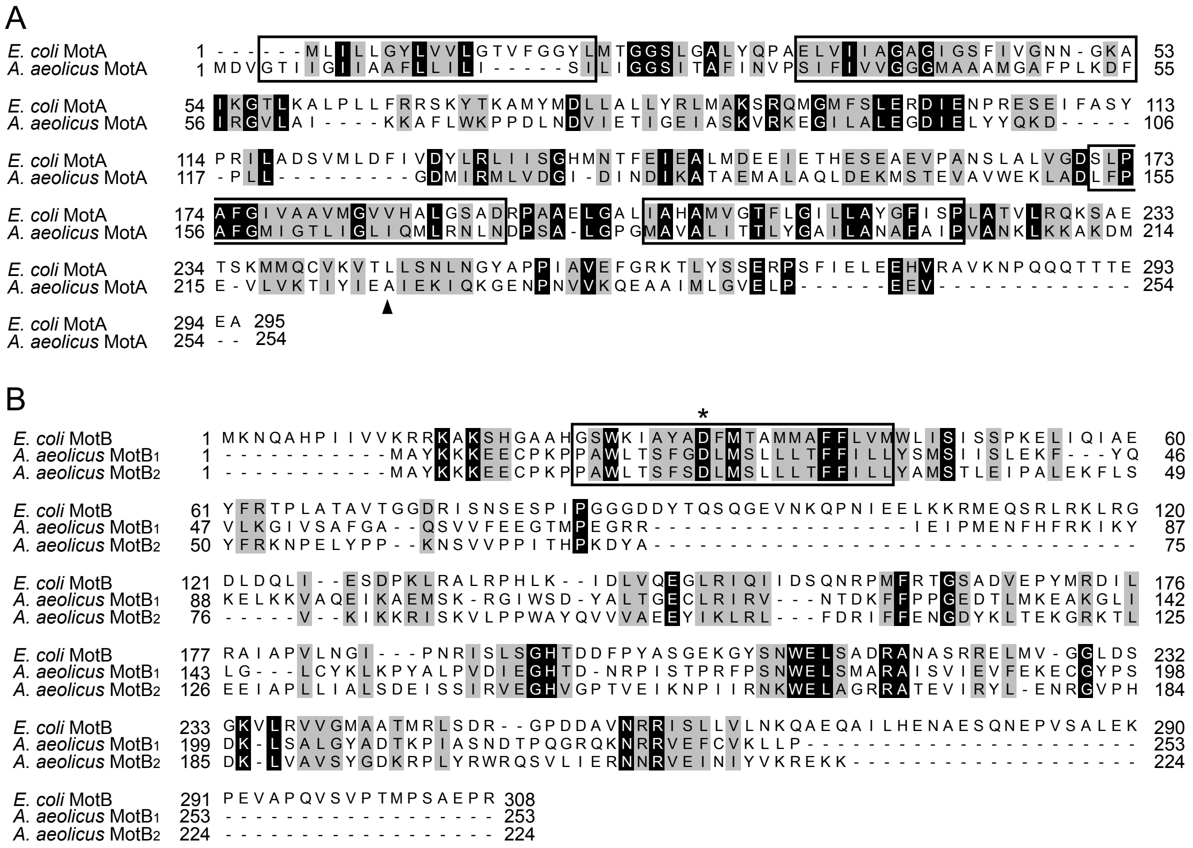

Fig. S2. Amino acid sequence alignments of MotA (A) and MotB (B) of *E. coli* and *A. aeolicus.* The MotA protein of *A. aeolicus* ($MotA^{Aa}$) shares 16.4 % sequence identity and 30.5% sequence similarity with MotA of *E. coli* ($MotA^{Ec}$). $MotB_1$ and $MotB_2$ of *A. aeolicus* ($MotB_1^{Aa}$ and $MotB_2^{Aa}$) share 27.3 % and 30.1 % sequence identity and 35.6% and 36.3% sequence similarity with MotB of *E. coli* ($MotB^{Ec}$), respectively. Identical residues are shown with a black background, and similar residues are shown with a gray background. The position of the up-motile mutation found in this study (MotA-A225) is marked with a black arrowhead. Putative TM segments are boxed with squares. *, conserved Asp residue binds coupling ion in MotB.

Fig. S2. Takekawa *et al.*

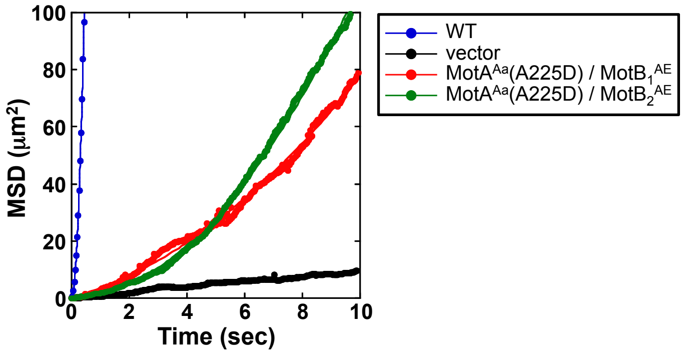

Fig. S3. MSD plot for *E. coli* cells producing the MotA and chimera MotB proteins. The movement of the *E. coli* cells was analyzed by calculating Mean Square Displacement (MSD) (*n* = 22 - 30). The non-motile cells laid a plot in proportion as time (black), showing that the cells made Brownian motion without any movement. The wild-type cells laid a plot in proportion as the square of time (blue), showing that the cells made directional motion. The chimeric cells laid a plot in proportion as both of time and the square of time (red and green), showing that the cells had very weak motility.

Fig. S3. Takekawa *et al.*

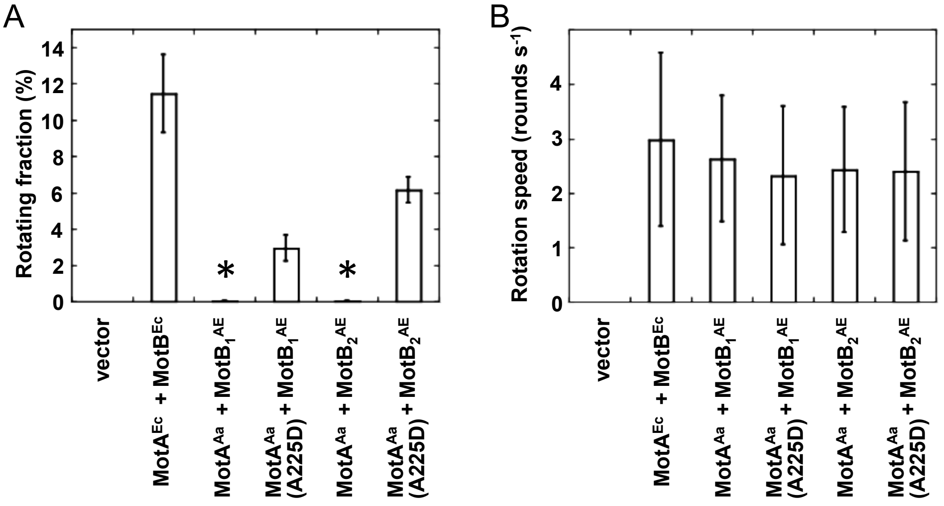

Fig. S4. Tethered cell assay of *E. coli* cells producing MotA and chimeric MotB proteins. (A) Rotating fractions of *E. coli* (Δ*motAB*) cells producing various stators. (B) Rotating Speeds of *E. coli* (Δ*motAB*) cells producing various stators. *; not 0 but less than 0.1 %.

Fig. S4. Takekawa *et al.*

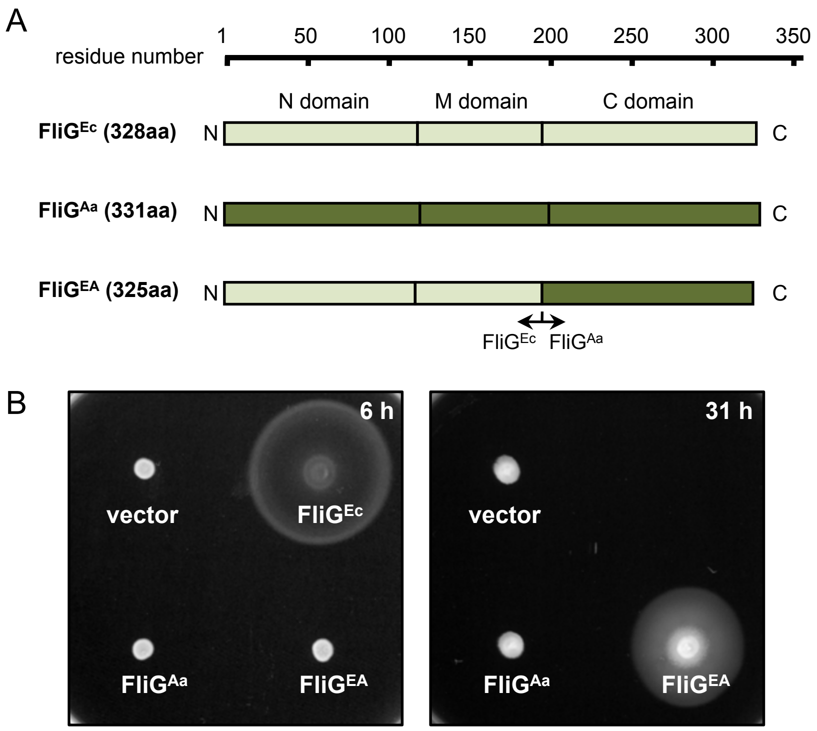

Fig. S5. Motility assay of *E. coli* cells producing chimeric FliG of *A. aeolicus* in soft-agar plate. (A) The schematics of primary structures of FliG of *E. coli* and *A. aeolicus* and chimera FliG. FliG is composed of three domain (N, M and C domain). We switched the sequence at the boundary of M and C domain for chimera FliG. (B) Wild-type FliG or chimeric FliG were expressed in a *E. coli* D*fliG* strain. Plates were incubated at 30°C for indicated hours in figures. ^{Ec}, protein of *E. coli*; ^{Aa}, protein of *A. aeolicus*; ^{EA}, chimera proteins fused proteins of *E. coli* and *A. aeolicus*.

Fig. S5. Takekawa *et al.*

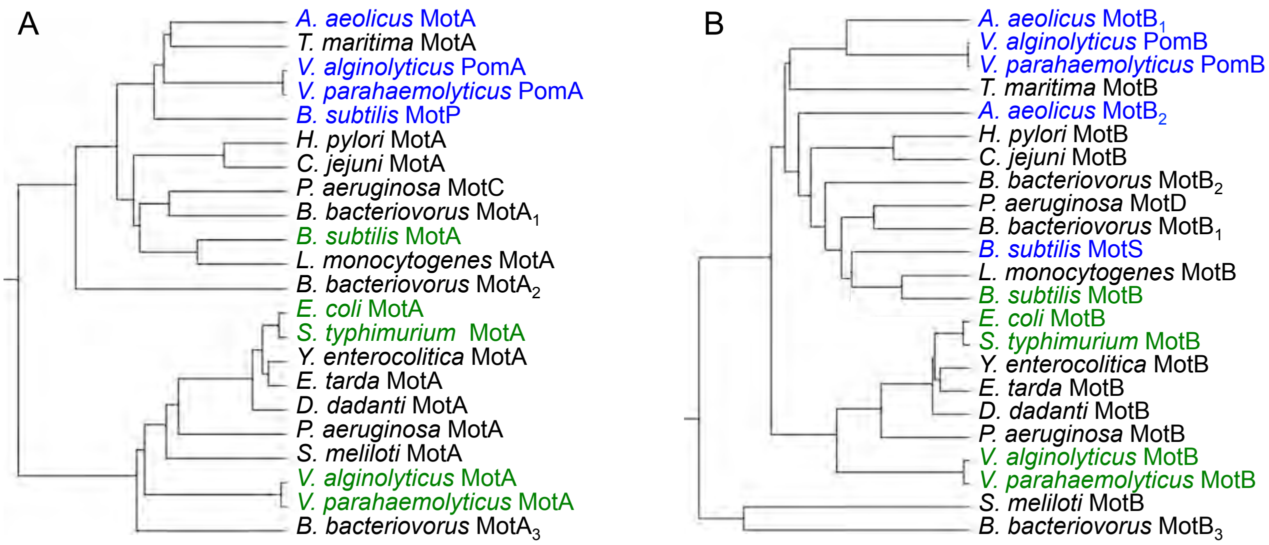

Fig. S6. Phylogenic tree of *motA*/*motB* from various bacteria. The phylogenic trees were drawn using the CLUSTALW program for *motA* genes (A) and for *motB* genes (B). Na^+-driven stators and the H^+-driven stators are indicated in blue and green, respectively.

Fig. S6. Takekawa *et al.*

Movie Legends

Movie S1. Swimming motility of *A. aeolicus* cells at 85°C. Scale bar in the move, 30μm

Movie S2. Swimming of *E. coli* cells. *E. coli* cells expressing chimeric stator ($MotA^{Aa}$(A225D)/$MotB_2^{AE}$) at room temperature (22°C) were observed.

Movie S3. Swimming of *E. coli* cells. *E. coli* cells expressing chimeric stator ($MotA^{Aa}$(A225D)/$MotB_2^{AE}$) at 45°C were observed.

Movie S4. Rotation of tethered *E. coli* cells. *E. coli* cells expressing chimeric stator ($MotA^{Aa}$(A225D)/$MotB_2^{AE}$) .
